# Estimating survival time of patients with glioblastoma multiforme and characterization of the identified microRNA signatures

**DOI:** 10.1186/s12864-016-3321-y

**Published:** 2016-12-22

**Authors:** Srinivasulu Yerukala Sathipati, Hui-Ling Huang, Shinn-Ying Ho

**Affiliations:** 1 Institute of Bioinformatics and Systems Biology, National Chiao Tung University, Hsinchu, Taiwan; 2 Department of Biological Science and Technology, National Chiao Tung University, Hsinchu, Taiwan

## Abstract

**Background:**

Though glioblastoma multiforme (GBM) is the most frequently occurring brain malignancy in adults, clinical treatment still faces challenges due to poor prognoses and tumor relapses. Recently, microRNAs (miRNAs) have been extensively used with the aim of developing accurate molecular therapies, because of their emerging role in the regulation of cancer-related genes. This work aims to identify the miRNA signatures related to survival of GBM patients for developing molecular therapies.

**Results:**

This work proposes a support vector regression (SVR)-based estimator, called SVR-GBM, to estimate the survival time in patients with GBM using their miRNA expression profiles. SVR-GBM identified 24 out of 470 miRNAs that were significantly associated with survival of GBM patients. SVR-GBM had a mean absolute error of 0.63 years and a correlation coefficient of 0.76 between the real and predicted survival time. The 10 top-ranked miRNAs according to prediction contribution are as follows: hsa-miR-222, hsa-miR-345, hsa-miR-587, hsa-miR-526a, hsa-miR-335, hsa-miR-122, hsa-miR-24, hsa-miR-433, hsa-miR-574 and hsa-miR-320. Biological analysis using the Kyoto Encyclopedia of Genes and Genomes (KEGG) pathway on the identified miRNAs revealed their influence in GBM cancer.

**Conclusion:**

The proposed SVR-GBM using an optimal feature selection algorithm and an optimized SVR to identify the 24 miRNA signatures associated with survival of GBM patients. These miRNA signatures are helpful to uncover the individual role of miRNAs in GBM prognosis and develop miRNA-based therapies.

**Electronic supplementary material:**

The online version of this article (doi:10.1186/s12864-016-3321-y) contains supplementary material, which is available to authorized users.

## Background

Glioblastoma multiforme (GBM) is the most common malignant human brain tumor [1]. There are two sub-types of glioblastoma, primary glioblastoma and secondary glioblastoma, which originate from different genetic pathways and affect patients of different ages [2]. Generally, standard therapies, such as radiotherapy and chemotherapy, do not contribute better survival benefits to GBM patients due to tumor reoccurrences even after multimodality treatment [3]. GBM patients’ median survival rate is very poor ranging from 12 to 14 months [4]. Early stage detection approaches are necessary to better understand the events in GBM and for the development of therapeutics.

MiRNA is a small (~18–22 nucleotides) non-coding RNA which targets messenger RNA (mRNA) for translation inhibition, thereby regulating protein expression [5]. MiRNA regulates several biological processes, such as cell proliferation [6], haematopoiesis [7], insulin secretion and apoptosis [8, 9]. Nowadays, miRNA expression profiling is extensively used in cancer studies due to its effective role in identifying cancer gene expression regulations. Many profiling studies have reported altered miRNA expressions in different cancers, including lung cancer, colon cancer, leukaemia, and glioblastoma [10–13]. Over the last several years, molecular characteristics have been used to predict tumor grades as well as to identify the microarrays which are associated with patient survival [14–16]. The combination of gene expression profiles and machine learning approaches have often been used to predict risk assessment, cancer recurrence and survivability, and to identify the potential biomarkers associated with cancer treatment. Gene expression profiling was used to identify genes which can classify different grades of tumors in GBM patients [17]. Fuller et al. used the microarray technology and k-nearest neighbour algorithm to classify tumor types in glioma patients [18]. Moreover, it was proven that miRNA expression profiles are more accurate in classifying different tumor types when compared with mRNA expression profiles [19]. Several studies reported that miRNA expression alterations have prognostic significance and are associated with overall survival among patients with GBM [20–22]. Recent miRNA-based studies revealed that miRNA expression is associated with chemo-resistance and radio-resistance [23, 24]. In conclusion, cancer treatment therapy based on miRNA expression profiles better contributes to the development of novel treatment and diagnosis approaches in patients with GBM.

Teplyuk et al. obtained promising accuracy using miRNA profiling of cerebrospinal fluid to develop a support vector machine model which distinguishes the glioblastoma and metastatic brain tumors [25]. Roth et al. distinguish glioblastoma patients from healthy controls using a support vector machine in order to identify the tumor-specific miRNAs and achieved an accuracy, sensitivity and specificity of 81, 83, and 79%, respectively [26]. A k-nearest neighbour method has been used to classify high-grade gliomas based on gene expression profiles and it was observed that the prediction models led to better clinical outcomes by separating diagnostically challenging malignant gliomas [27]. Current studies of prediction methods have used small datasets and the majority of proposed methods are concerned with detection and classification of different types of tumors and malignancies.

However, before miRNA expression profiling can be implemented in clinical practice, effective methods which can be applied to large datasets are still needed for the development of potential therapeutics associated with patients’ survival. Accordingly, this work proposes a support vector regression (SVR)-based method, called SVR-GBM, for identification of miRNAs to estimate the survival time in patients with GBM. High performance of SVR-GBM was derived from an optimal feature selection method, inheritable bi-objective combinatorial genetic algorithm (IBCGA) [28]. In this work, we utilized the cancer genome atlas (TCGA) data portal to obtain miRNA expression profiles of 247 patients with GBM. SVR-GBM identified 24 out of 470 miRNAs for the prediction of survival time in patients with GBM and obtained a mean absolute error of 0.63 years and a correlation coefficient of 0.76 between the real and predicted survival time. Further, we ranked these miRNAs based on their contribution to the survival time prediction. The biological significance of the 10 top-ranked miRNAs in cancer pathways was analysed. The identified miRNA signatures may help to develop miRNA-based therapies in GBM medicine.

## Results and Discussion

### Estimation of survival time

**Fig. 1:**
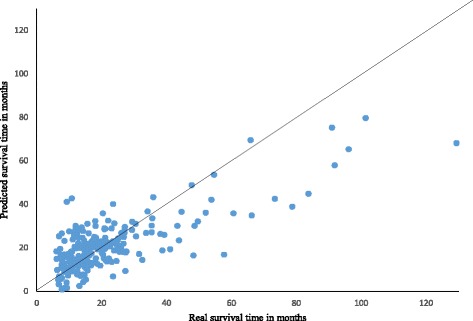
Predicted survival time on Y-axis and real survival time on X-axis

We made an attempt to estimate survival time of GBM cancer patients using their miRNA expression profiles. We utilized 247 patients with GBM and the survival time of these patients was between 0.4 to 11 years. SVR-GBM used an optimal feature selection algorithm IBCGA to identify 24 out of 470 miRNAs which are associated with survival time of cancer patients. This study is the first to use a support vector regression model combining with an optimal feature selection of miRNAs to estimate survival time among patients with GBM. SVR-GBM achieved a correlation coefficient of 0.76 and a mean absolute error of 0.63 years using 10-fold cross-validation. The correlation plot between real and predicted survival time is shown in Fig. 1.

We employed multiple regression analysis using the stepwise feature addition method [29] and elastic net [30] to compare with SVR-GBM. The comparison results are shown in Table 1. SVR-GBM achieved a correlation coefficient, mean absolute error, and standard error of estimates of 0.76, 0.63 years and 11.34, respectively; better than the multiple linear regression with the correlation coefficient, mean absolute error, and standard error of estimates of 0.63, 0.80 years and 13.97, respectively; and the elastic net method with the correlation coefficient, mean absolute error, and standard error of estimates of 0.39, 0.86 years and 16.35, respectively.

**Table 1:** Prediction performance of SVR-GBM

Method	Features selected	Correlation coefficient	Mean absolute error *(MAE)*	Standard error of estimates
SVR-GBM	24	0.76	0.63	11.34
Multiple regression analysis	15	0.63	0.80	13.97
Elastic net	6	0.39	0.86	16.35

### Ranks of the identified miRNA signatures

We performed a main effect difference (MED) analysis to reveal the contribution of each miRNA to the survival prediction model by an orthogonal experimental design [31]. The 24 identified miRNAs and MED scores are shown in Table 2. The 10 top-ranked miRNAs using the MED analysis are hsa-miR-222, hsa-miR-345, hsa-miR-587, hsa-miR-526a, hsa-miR-335, hsa-miR-122, hsa-miR-24, hsa-miR-433, hsa-miR-574, and hsa-miR-320. Furthermore, we assessed the biological significance of these 10 miRNAs using the KEGG pathway analysis.

**Table 2:** Results of the main effect difference analysis. 24 miRNA sequences and corresponding MED scores

miRNA	MED	Mature sequence
hsa-miR-222	0.796768	AGCUACAUCUGGCUACUGGGU
hsa-miR-345	0.567302	GCUGACUCCUAGUCCAGGGCUC
hsa-miR-587	0.535874	UUUCCAUAGGUGAUGAGUCAC
hsa-miR-526a	0.461675	CUCUAGAGGGAAGCACUUUCUG
hsa-miR-335	0.457645	UCAAGAGCAAUAACGAAAAAUGU
hsa-miR-122	0.443237	UGGAGUGUGACAAUGGUGUUUG
hsa-miR-24	0.427016	UGGCUCAGUUCAGCAGGAACAG
hsa-miR-433	0.40424	AUCAUGAUGGGCUCCUCGGUGU
hsa-miR-574	0.338432	CACGCUCAUGCACACACCCACA
hsa-miR-320	0.337497	AAAAGCUGGGUUGAGAGGGCGA
hsa-miR-768	0.304775	GUUGGAGGAUGAAAGUACGGAGUGAU
hsa-miR-223	0.287394	CGUGUAUUUGACAAGCUGAGUU
hsa-miR-497	0.266012	CAGCAGCACACUGUGGUUUGU
hsa-miR-370	0.220475	CAGGUCACGUCUCUGCAGUUAC
hsa-miR-137	0.219401	UUAUUGCUUAAGAAUACGCGUAG
hsa-miR-605	0.210376	UAAAUCCCAUGGUGCCUUCUCCU
hsa-miR-491	0.207076	AGUGGGGAACCCUUCCAUGAGG
hsa-miR-656	0.204699	AGGUUGCCUGUGAGGUGUUCA
hsa-miR-15b	0.170935	UAGCAGCACAUCAUGGUUUACA
hsa-miR-801	0.170311	GAUUGCUCUGCGUGCGGAAUCGAC
hsa-miR-221	0.129155	ACCUGGCAUACAAUGUAGAUUU
hsa-miR-95	0.104175	UCAAUAAAUGUCUGUUGAAUU
hsa-miR-603	0.099838	CACACACUGCAAUUACUUUUGC
hsa-miR-519c	0.02755	CUCUAGAGGGAAGCGCUUUCUG

### Characteristics of the identified miRNAs

1. Hsa-miR-222: This miRNA plays a critical role in GBM intervention. Hsa-miR-221/222 are often upregulated in GBM. This miRNA regulates cell proliferation in U251 glioma cells by targeting the functional p27kip1 gene (a member of the kip family of cyclin-dependent kinase inhibitors) [32], and co–suppression of this miRNA by the antisense approach inhibits advanced tumor cell proliferation and may function as a potential therapeutic in glioma [32]. Zhang et al. found the inverse relation between mir-222 and pro-apoptotic genes in glioma cells [33]. Alteration of this miRNA in glioma cells upregulates PUMA expression and promotes apoptosis, thus reducing tumor size [33]. In addition, investigation of glioma cell lines revealed that hsa-miR-222 also targets the gene TIMP2, suppression of this miRNA regulated cell invasion and angiogenesis [34]. Experimental validation in malignant glioma cells concluded that mir-222 acts as an oncogenic by targeting connexin 43 (Cx43) and regulating cell proliferation and invasion [35]. Moreover, mir-222 plays an important role in small cell lung cancer and hepatocellular carcinoma by targeting phosphate and tensin homolog and the tissue inhibitors of metallo-proteinase tumor suppressors and by enhancing cellular migration [36].
2. Hsa-miR-345: Zinn et al. reported that hsa-miR-345 was correlated with short survival times in glioblastoma patients [37]. We observed that though the participation of hsa-miR-345 is limited in glioblastoma, it’s expression is often deregulated in other major cancer types. For instance, hsa-miR-345 has been found to be deregulated in non-small cell lung cancer, and its expression is associated with clinicopathelogical features [38]. In prostate cancer, mir-345 regulates cell proliferation, invasion, and migration by targeting the Smad1 gene [39]. Luciferase assay analysis reported that BCL-2 associated anthanogene-3 is the target of mir-345, and over expression of this miRNA suppresses cell proliferation and invasion in colorectal cancer cells in vitro [40].
3. Hsa-miR-335: A real-time quantitative RT-PCR assay study reported that the expression of hsa-miR-335 is significantly associated with the clinicopathelogical factors and survival time of patients with GBM. It was also noted that expression levels of mir-335 were higher in a short survival group, when compared with a long survival group [41]. In most cases, it was down-regulated in breast and ovarian cancers. In breast cancer cell lines, mir-335 targets three prime untranslated regions of c-Met and subsequently inhibits cell migration [42]. Mir-335 expression is down-regulated in ovarian cancer cell lines when compared with adjacent normal counterparts [43]. In neuroblastoma, mir-335 regulates the transforming growth factor-β (TGF-β) non-canonical pathway and inhibits the transient potential of neuroblastoma cells [44].
4. Hsa-miR-24: A qRT-PCR assay study reported that hsa-miR-24 acts as an oncogene that directly targets ST7L and suppresses the β-catenin/ Tcf 4 transcription activity, and that further suppression of this miRNA expression regulates cell proliferation and invasion in glioma cells [45]. MTT assay analysis revealed that hsa-miR-24 targets the MXI1 tumor suppressor gene and promotes cell proliferation, and that it is upregulated in glioma cells [46]. Upregulation of mir-24 was also observed in breast and non-small cell lung cancers. In breast cancer, mir-24 directly targets the p27Kip1 and inhibits apoptosis in MDA-MB-435 and MDA-MD-468 cells [47], as well as in non-small cell lung cancer cells. This miRNA targets nuclear apoptosis–inducing factor 1 and induces cell proliferation [48].
5. Hsa-miR-320: Quantitative real-time PCR analysis was used to assess human glioma cell lines and it was reported that expression of hsa-miR-320a correlated with patient prognoses. Its over- expression regulates the insulin-like growth factor-1 receptor and acts as a tumor-suppressor in glioma [49]. Lower expressions of hsa-miR-320 were observed when compared with healthy brain tissues, and also over expression of this miRNA inhibits cell proliferation and metastasis by targeting the cell cycle regulator E2F1 [50]. Most often, down regulation of mir-320 was observed and functioned as a potential biomarker for early stage detection in colorectal carcinoma [51].

While the remaining miRNAs in the top-ranked miRNA list, hsa-miR-587, hsa-miR-526a, hsa-miR-122, hsa-miR-433, and hsa-miR-574 (scored 0.53, 0.46, 0.44, 0.40 and 0.33 respectively), were not directly involved in GBM, they are, with one exception, actively associated with the major cancer types and diseases. Though, they have less experimental validations in glioblastoma, their contribution towards the survival estimation is high according to the MED analysis. Hsa-miR-526a inhibits apoptosis in tumor cells by targeting the CYLD, and plays a potential role in tumor migration and invasion via the NF-kB signaling pathway [52]. Hsa-miR-122 is frequently down-regulated in hepatocellular carcinoma, which targets peroxiredoxin 2 and induces apoptosis [53]. Hsa-miR-433 is down-regulated and is a target of tumor associated proteins GRB2 and RAB-94 in gastric cancer [54]. Hsa-miR-574 is involved in the suppression of colorectal cancer liver metastasis by negatively regulating the metastasis associated in colon cancer [55]. The lone member of the top-10 miRNA not previously associated with cancer types or diseases is hsa-miR-587. The membership on this list indicates that hsa-miR-587 may be a valuable subject of further exploration. Although these top-ranked miRNAs do not directly participate in the glioblastoma cancer, they are worthy subjects for further investigation in GBM cancer and might help in the gene target based therapies.

Besides the 10 miRNAs listed in the main effect difference results table (Table 2), several of the 14 other identified miRNAs, such as hsa-miR-223, hsa-miR-497, hsa-miR-137, hsa-miR-656 and hsa-miR-221 (scored 0.28, 0.26, 0.21 and 0.20 respectively), have also been found to play a potential role in GBM progression. Hsa-miR-223 targets the paired box 6 (PAX6), which regulates proliferation and invasion of glioblastoma cells [56]. Hsa-miR-497 expression was associated with glioma drug resistance and it acts as a potential molecular target in glioma cells [57]. Hsa-miR-137 plays a key role in glioma, often it was downregulated. Recent investigation indicated that direct overexpression of hsa-miR-137 and delphinidin treatment effectively controlled glioblastoma growth [58]; this miRNA also induces apoptosis and inhibits the growth of glioma cells by targeting RAC1 [59]. Hsa-miR-656 expression levels are downregulated in glioma, and it inhibits the neurosphere formation and cell proliferation in glioma cell lines by targeting the bone morphogenetic protein −2 receptor, type-1 A (BMPR1A) [60]. Expression levels of hsa-miR-221 in glioma are significantly upregulated; mir-221/222 module regulates cell proliferation and apoptosis in glioma cell lines by targeting PUMA and suppressing tumor size [32, 33]. Hsa-miR-603 stimulates cell proliferation via β-catenin-interacting protein 1 (CTNNBIP1) and Wnt inhibitory factor 1 (WIF1) in glioma cell lines in vitro and in vivo [61].

It is the work’s finding that the set of the 24 miRNA signatures can be used to estimate the survival time in patients with GBM. Additionally, the 10 top-ranked miRNAs contributed well towards survival estimation and analysis of these miRNAs revealed their functionality in various properties of cancer cell, such as proliferation, invasion and apoptosis, which can assist the understanding of mechanism of cancer progression in GBM. Several miRNAs in our study have been directly observed participating in GBM; however, a few miRNAs are not directly implicated in GBM, but they contributed towards survival estimation and many also play a key role in other major cancer types.

**Fig. 2:**
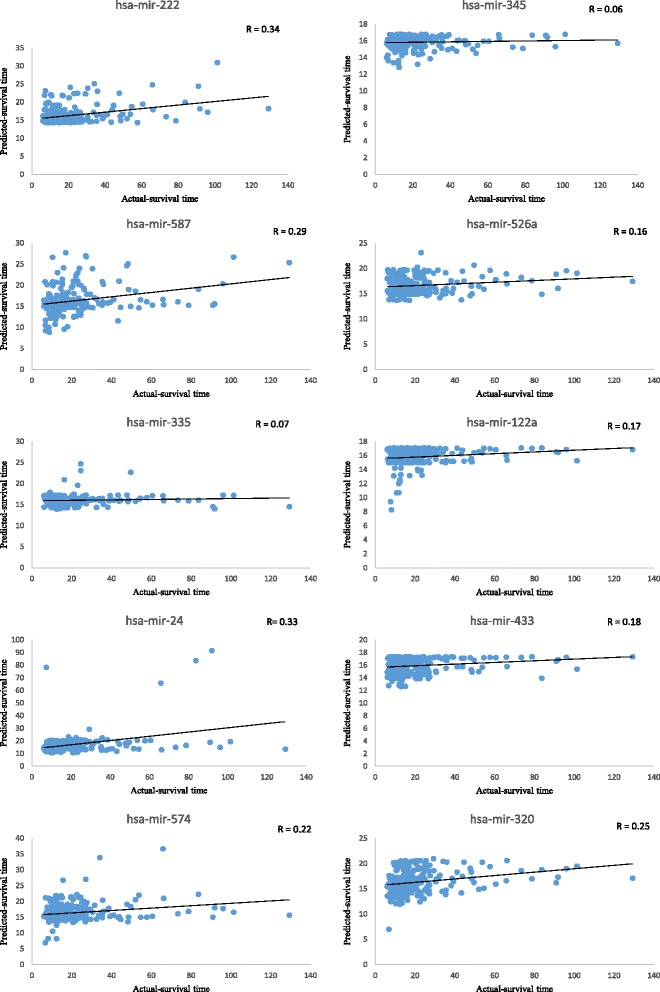
Individual effect of miRNA on survival time estimation. Top-10 miRNA correlation plots

To measure the individual effect of these 24 identified miRNAs on survival time estimation, we used feature knock-out analysis. The 10 miRNAs, hsa-miR-222, hsa-miR-345, hsa-miR-587, hsa-miR-526a, hsa-miR-335, hsa-miR-122, hsa-miR-24, hsa-miR-433, hsa-miR-574, and hsa-miR-320, individually contributed correlation coefficients of 0.34, 0.06, 0.29, 0.16, 0.07, 0.17, 0.33, 0.18, 0.22, and 0.25 respectively corresponding mean absolute error is also shown in Table 3. Correlation plots for the 10 top-ranked miRNAs are shown in Fig. 2. The remaining 14 miRNAs among the 24 are shown in Additional file 1: Figure S1.

**Table 3:** Individual effects of miRNAs on survival estimation

miRNAs	Correlation coefficient	Mean absolute error (in months)
hsa-miR-222	0.34	9.58
hsa-miR-345	0.06	9.73
hsa-miR-587	0.29	9.24
hsa-miR-526a	0.16	9.65
hsa-miR-335	0.07	9.65
hsa-miR-122	0.17	9.61
hsa-miR-24	0.33	8.76
hsa-miR-433	0.18	9.59
hsa-miR-574	0.22	9.33
hsa-miR-320	0.25	9.32

### KEGG pathway

**Fig. 3:**
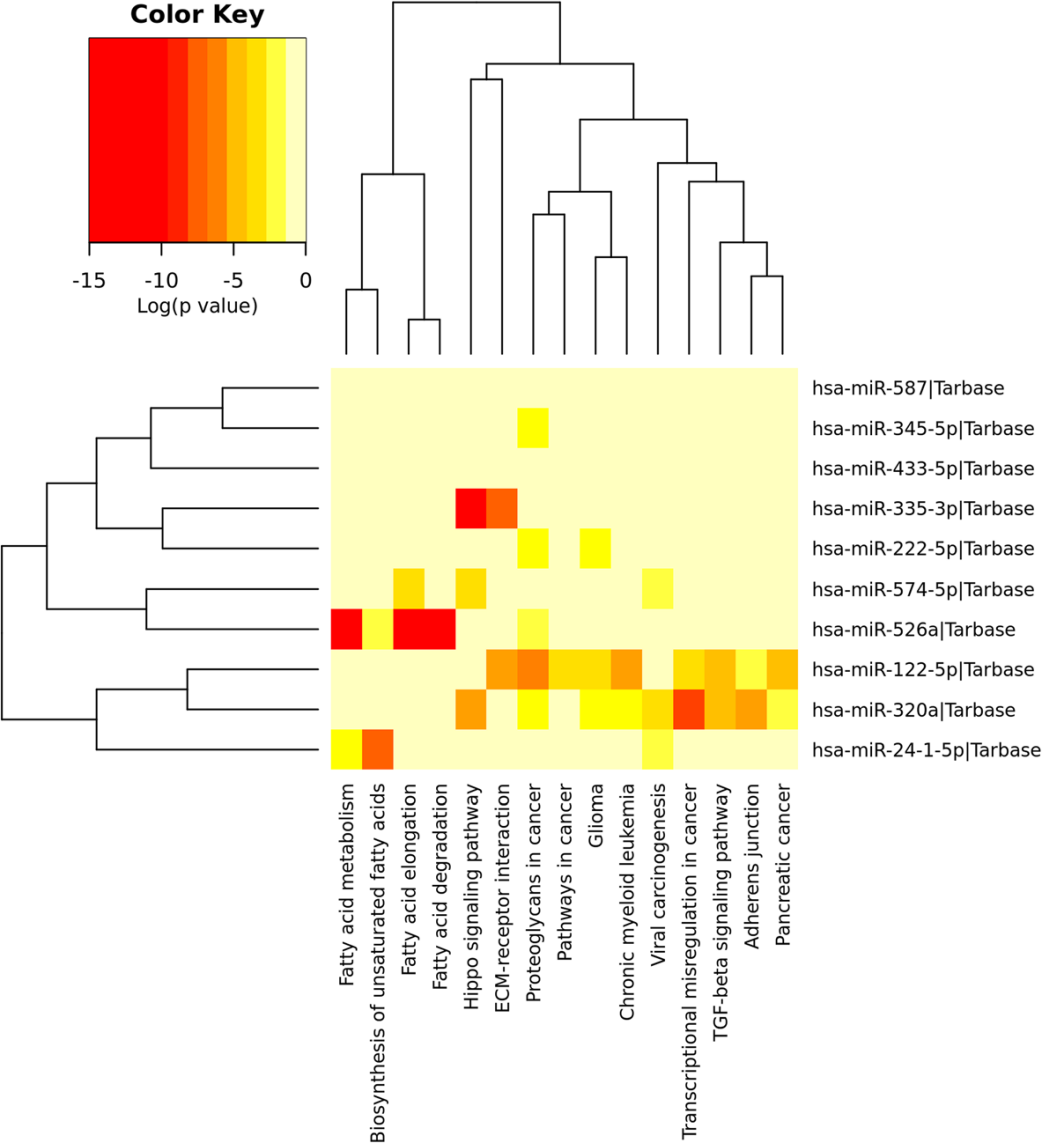
Heat map of the KEGG pathway. 10 miRNA signatures involved in different cancer pathways including glioma and signaling pathways

To evaluate the biological significance of the 24 identified miRNAs involved in cancer and non-cancer pathways, we employed the KEGG pathway analysis using the DIANA tools. The 10 top-ranked miRNAs show statistical significance with cancers, such as chronic myeloid leukemia, glioma, pancreatic cancer, non-small cell lung cancer, colorectal cancer melanoma, and prostate cancer, and signaling pathways, such as Hippo signaling pathway, TGF-beta signaling pathway, thyroid hormone signaling pathway, FoxO signaling pathway, and mRNA surveillance pathway to name a few. Complete KEGG pathway analysis of these 10 miRNAs and statistical significance in different pathways and number of involved genes are shown in Table 4. The 10 top-ranked miRNAs and their target gene enrichment in cancer and signaling pathways are shown in Fig. 3 and all the 24 miRNAs gene enrichment analysis is shown in Additional file 1: Figure S2.

**Table 4:** The 10 top-ranked miRNAs and their target gene involvement in the KEGG pathway

KEGG pathway	Genes	miRNAs	*p*-value
Hippo signaling pathway	33	3	1.72E-12
Fatty acid elongation	3	2	5.35E-12
Proteoglycans in cancer	60	5	1.36E-08
Fatty acid metabolism	2	2	1.79E-08
Transcriptional misregulation in cancer	55	2	1.40E-06
ECM-receptor interaction	16	2	1.71E-06
Fatty acid degradation	1	1	3.97E-06
Chronic myeloid leukemia	33	2	0.00026795
Glioma	25	3	0.00035641
TGF-beta signaling pathway	29	2	0.00181306
Adherens junction	27	2	0.00272898
Biosynthesis of unsaturated fatty acids	2	2	0.00488867
Viral carcinogenesis	50	3	0.01025578
Pathways in cancer	59	1	0.03687983
Pancreatic cancer	26	2	0.03809962
Metabolism of xenobiotics by cytochrome P450	2	1	0.05544785
Signaling pathways regulating pluripotency of stem cells	43	2	0.06508793
Central carbon metabolism in cancer	24	2	0.06577197
Non-small cell lung cancer	15	1	0.1204275
Colorectal cancer	23	2	0.129777
Thyroid hormone signaling pathway	42	2	0.1389078
Other types of O-glycan biosynthesis	8	1	0.2209965
Lysine degradation	4	1	0.223509
Spliceosome	24	2	0.2736693
Small cell lung cancer	23	1	0.2798967
Prostate cancer	26	2	0.3062628
Melanoma	22	2	0.3099633
Insulin signaling pathway	31	1	0.3635885
Antigen processing and presentation	5	1	0.4749925
Shigellosis	6	1	0.5180352
Cell cycle	22	1	0.5701685
Steroid biosynthesis	1	1	0.6006251
FoxO signaling pathway	25	1	0.6250634
Sulfur relay system	2	2	0.6482195
Estrogen signaling pathway	19	1	0.6860049
Long-term depression	10	1	0.6946705
Base excision repair	2	1	0.738435
Protein processing in endoplasmic reticulum	10	1	0.7774063
mRNA surveillance pathway	7	1	0.8486927
RNA transport	29	1	0.8553475
AMPK signaling pathway	23	1	0.859823
Huntington’s disease	2	1	0.9555257
Adipocytokine signaling pathway	13	1	0.9654451
Allograft rejection	3	1	0.9744973
Cocaine addiction	8	1	0.9772773
Purine metabolism	5	1	0.9896283
Renin-angiotensin system	1	1	0.9943529
Valine, leucine and isoleucine degradation	1	1	0.9945874
Valine, leucine and isoleucine biosynthesis	1	1	0.9973641

### Target gene prediction

After identifying the miRNAs associated with survival time, we conducted target gene prediction for the set of 10 top-ranked miRNAs using miRTarBase [62]. We identified 162 non-redundant experimentally strong evidence target genes for hsa-miR-222, hsa-miR-345, hsa-miR-335, hsa-miR-24, hsa-miR-433, hsa-miR-574, and hsa-miR-320 (data not shown). MiRNAs act as both tumor suppressors and oncogenes in different cancer pathways for these target genes. So, we reported the participation of each miRNA in different cancer types. Among the 10 miRNAs, seven miRNAs have experimentally validated genes and their regulation in various cancer types. Experimentally validated genes and miRNA regulation are shown in Table 5.

**Table 5:** Experimentally validated target genes for miRNAs

miRNA	Target gene	Regulation	Validation method	Cancer	Reference
hsa-miR-222	GJA1	down	Immunofluorescence, Western Blot, Luciferase Reporter Assay	Glioblastoma	[35]
	CDKN1C	down	Luciferase Reporter Assay	Glioblastoma	[69]
	P27kip1	down	Western Blot	Glioblastoma	[32]
	DICER1	down	Luciferase Reporter Assay	Breast cancer	[70]
	TIMP3	Down	Luciferase Reporter Assay, qPCR, Western Blot	Lung cancer	[36]
	CDKN1B	Down	Luciferase Reporter Assay	Thyroid carcinoma	[71]
	PPP2R2A	Down	Luciferase Reporter Assay	Lung cancer	[72]
hsa-miR-345	MCL-1 and BCL2L2	Up	microarray	Lung cancer	[73]
	Smad1	Down	microarray	Prostate cancer	[39]
	BAG3	Down	Luciferase reporter assay and western blot	colorectal cancer	[40]
hsa-miR-335	SOX4	Down	Northern blot, qRT-PCR etc.	breast cancer	[74]
	TGF-β	Down	qRT-PCR	Neuroblastoma	[44]
hsa-miR-122a	CCNG1	Down	Northern blot, qRT-PCR	hepatocellular carcinoma	[75]
hsa-miR-24	ST7L	Up	qRT-PCR	glioma	[45]
	MXI1	Up	MTT assay	glioma	[46]
hsa-miR-433	FGF20	-	- Northern blot, qRT-PCR etc.	Parkinson’s disease	[76]
hsa-miR-320	TfR-1	Down	northern blot, qRT-PCR	acute myeloid leukemia	[77]
	Mcl-1, BCL2	Down	northern blot, qRT-PCR	cholangiocarcinoma	[78]

## Conclusion

This study presents the identification of miRNA signatures with respect to their correlation with survival time in patients with GBM. Many studies used the GBM data from the TCGA data portal. However, the outcome results were accordingly not the same. In fact, the extracted miRNA profiles based on clinical follow up and filtered procedures were different across all the studies. In this work, we first developed a miRNA expression profile-based survival time estimation method called SVR-GBM, which incorporates the optimal feature selection algorithm IBCGA. SVR-GBM identified 24 miRNAs associated with the survival time in patients with GBM. Our model estimated the survival time of 247 patients with GBM and achieved a correlation coefficient of 0.76 and a mean absolute error of 0.63 years, and is comparatively better than multiple regression analysis method. In this work, miRNA expression profiles were solely used to estimate the survival time, the results were not tremendous. The model can be refined by considering other factors, such as mRNA and protein expression profiles. Furthermore, we ranked the 24 identified miRNAs based on their contribution towards the survival time estimation. The biological significance of these miRNAs was discussed, and miRNA analysis revealed their functional role in GBM cancer and other major cancer types. This study would provide a new insight into molecular therapeutic approaches to improving the therapies of GBM patients.

## Methods

### Dataset

All miRNA expression profiles of glioblastoma patients and corresponding clinical information were retrieved from the TCGA database. Level 3 data of 528 samples on the Agilent human 8X15k were downloaded. We followed certain criteria to retrieve samples: (i) the patients who undergone chemotherapy/radiotherapy, (ii) the patients who had survival information (days to death), (iii) the patients whose survival period equal or greater than 30 days, and (iv) elimination of duplicate entries by merging all patient lists and the corresponding survival periods. After filtering out the samples, there were a total of 247 samples with 470 miRNAs, which we used for further analysis.

### SVR-GBM

We proposed a novel method SVR-GBM to predict the survival time in patients with GBM. This method also identifies the informative miRNAs to determine their functionality in GBM.

### Integration of IBCGA and SVR for miRNA selection and modeling

Support vector machine (SVM) is based on statistical learning theory and successfully applied to classification and regression problems [63]. In this work, we approached support vector regression method to estimate the survival time in patients with GBM. The ν support vector regression (SVR) presents the good performance because it relies on the number of support vectors and training error. Given a set of data points, (*x*_*1*_, *y*_*1*_), (*x*_*2*_ *, y2*)… (*x*_*m*_ *,y*_*m*_), where *x*_*i*_ ∈ *R* ^*n*^ is an input and *y*_*i*_ ∈ *R* ^*1*^ is a target output. The optimization problem of the ν-SVR can be defined as follows.

1 $$ min\left[\right\{\frac{1}{2}{w}^T\left(\phi \left({x}_i\right)+b\right)+C\left(\upsilon \kern0.5em \varepsilon +\frac{1}{m}{\displaystyle \Big({\sum}_{i=1}^m\kern0.5em \left({\xi}_i+{\xi}_i^{*}\right)\left)\right)}\right\}\Big] $$

where *ξ*_*i*_ ≥ 0, *ξ*_i_^*^ ≥ 0, ε ≥ 0; i = 1, 2, …, m; and b is a constant.

Here, 0 ≤ ν ≤ 1, and *C* is the regularization parameter. The ε-insensitive loss function means that if *w* ^*T*^ ∅ (*x*_*i*_) is in the range of y ± ε no loss is considered. The y ± ε is as known as the soft margin where ν is an upper bound on the fraction of margin errors and a lower bound of the fraction of support vectors . In this work, the LibSVM package was used for implementation ofν-SVR [64]. To select a minimal set of informative features from a large number of candidate features problem, the inheritable bi-objective combinatorial genetic algorithm (IBCGA) [28] was used. In this work, we incorporated the optimal feature selection algorithm IBCGA and ν-SVR to obtain an optimized model. The parameters for designing the SVR model to be optimised simultaneously by the IBCGA are the *n* binary variables for selecting informative miRNAs and tuning parameters *C*, γ and ν of the SVR. The chromosome of the IBCGA comprises *n* binary genes *f*_i_ to select *m* miRNA and three 4-bit genes for encoding γ, C, and ν of the SVR. The i-th miRNA is excluded from the SVR regression model if *f*_*i*_ = 0 and included if *f*_*i*_ = 1. The sum of *f*_*i*_ is equal to *m*. The IBCGA can simultaneously obtain a set of solutions, *X*_*r*_ *,* where *r* = *r*_*start*_, *r*_*start*_ + 1, …, *r*_*end*_ in a single run. In this work, the parameter values are *r*_*start*_ = 10, *r*_*end*_ = 50, *N*_*pop*_ = 50, *P*_*c*_ = 0.8, *P*_*m*_ = 0.05, and *Gmax* = 60 [28].

To maximize the estimation accuracy in terms of Pearson’s correlation coefficient (*r*) used as the fitness function, we employed 10-fold cross validation (10-CV) to measure the performance of SVR-GBM in terms of Pearson’s correlation coefficient and mean absolute error between the predicted survival time and real survival time.

The Pearson’s correlation coefficient (*r*) can be formulated as follows

2 $$ r=\frac{{\displaystyle {\sum}_{i=1}^N}\left({x}_i-\overline{x}\right)\ \left({y}_i-\overline{y}\right)}{\sqrt{\left\lfloor {\displaystyle {\sum}_{i=1}^N}{\left({x}_i-\overline{x}\right)}^2\right\rfloor \left[{\displaystyle {\sum}_{i=1}^N}{\left({y}_i-\overline{y}\right)}^2\right]}} $$

where *x*_i_ and *y*_i_ are real and predicted survival time of the *i* ^th^ miRNA, and $\overline{x}$ and $\overline{y}$ are their corresponding means. Here *N* is the total number of miRNAs in the sample.

The mean absolute error (*MAE*) is described by

3 $$ MAE = \frac{1}{N}{\displaystyle \sum_{i=1}^N}{\left|{y}_i-{x}_i\right|}^2 $$

The standard error of estimates (*SEE*) is defined as

4 $$ SEE = \sqrt{\frac{{\displaystyle \sum {\left({y}_i-{x}_i\right)}^2}}{n-2}} $$

where *y*_i_ is estimated value and *x*_i_ is actual value, *n* is number of observations.

The customised IBCGA is described below.

Step 1) (Initialization) Generate an initial population of *N*_*pop*_ individuals randomly. All the *n* binary genes *f*_i_ have *r* 1’s and *n*-*r* 0’s, where *r* = *r*_*start*_.
Step 2) (Evaluation) Evaluate all individuals using the fitness function.
Step 3) (Selection) Use a tournament selection method that selects the winner from two randomly selected individuals to form a mating pool.
Step 4) (Crossover) Select *P*_*c*_ · *N*_*pop*_ parents from the mating pool to perform the orthogonal array crossover, where *P*_*c*_ is the crossover probability.
Step 5) (Mutation) The traditional mutation operator is applied to the randomly selected *P*_*m*_ · *N*_*pop*_ individuals except the best individual, where *P*_*m*_ is the mutation probability.
Step 6) (Termination test) If the stopping condition of performing *G*_*max*_ generations for obtaining the solution *X*_*r*_ is satisfied, output the best individual as *X*_*r*_. Otherwise, go to Step 2.
Step 7) (Inheritance) If *r* < *r*_*end*_, randomly change one bit in the binary genes *f*_*i*_ for each individual from 0 to 1; increase the number *r* by one, and go to Step 2. Otherwise, stop the algorithm.
Step 8) (Output) Let *m* equal the value of *r* having the best fitness value. Output the *m* miRNAs and the corresponding SVR model.

### Multiple linear regression

We employed a multiple regression technique to estimate the survival time. Stepwise feature addition procedure has been used for feature selection. In multiple linear regression, every value of the independent variable *x* is associated with the dependent variable value *y* [65]. A general multiple linear regression can be defined as

5 $$ {y}_i={\beta}_0 + {\beta}_1{x}_1 + {\beta}_2{x}_2+\cdots +{\beta}_n{x}_n + \varepsilon . $$

where *y*_*i*_ is the dependent variable; *x*_*1*_, *x*_*2*_, …, *x*_n_ are the independent variables; *β*_*0*_, *β*_*1*_, *β*_*2*_, *…*, *β*_*n*_ are the regression coefficients; *n* denotes the number of terms in the model, and Ɛ is the error term.

### Diana tools

We employed miRNA pathway analysis using DIANA-mirpath webserver [66] which utilized DIANA-Tarbase algorithm to predict the miRNA target. In order to estimate the specificity of results, we performed the pathway analysis for all identified miRNAs. In the mirpath tool, we selected the pathway union feature to identify the specific targeted KEGG pathway for each identified miRNA. The mirpath server employs enrichment analysis and measures the significance levels (p-value) between identified miRNAs and corresponding pathways using Fisher’s exact test. The results of this analysis indicate that the probability of particular pathway is notably enriched with targeted by at least one selected miRNA.

### Gene Target prediction

We used miRTarBase [62] and Tarbase [67] to predict the experimentally validated gene targets. Mir2 disease [68] was used to identify the cancer related miRNAs.

## Additional file

Additional file 1: Additional file contains the following Figures and Tables. **Figure S1.** Individual effect of miRNA on survival time estimation. Correlation plots for 14 miRNAs **Figure S2.** Heat map of the KEGG pathway. Identified 24miRNA signatures involved in different cancer pathway and signaling pathways. **Table S1.** 24 miRNAs and their gene enrichment in the KEGG pathway. (PDF 360 kb)
