# Supplementary material for: Estimating survival time of patients with glioblastoma multiforme and characterization of the identified microRNA signatures
Source: BMC Genomics. 2016 Dec 22;17(Suppl 13):1022. doi: 10.1186/s12864-016-3321-y (PMC5260001; doi:10.1186/s12864-016-3321-y)
Supplement: Additional file 1: — Additional file contains the following Figures and Tables. Figure S1. Individual effect of miRNA on survival time estimation. Correlation plots for 14 miRNAs Figure S2. Heat map of the KEGG pathway. Identified 24miRNA signatures involved in different cancer pathway and signaling pathways. Table S1. 24 miRNAs and their gene enrichment in the KEGG pathway. (PDF 360 kb) [file 12864_2016_3321_MOESM1_ESM.pdf]

Supplementary file 1

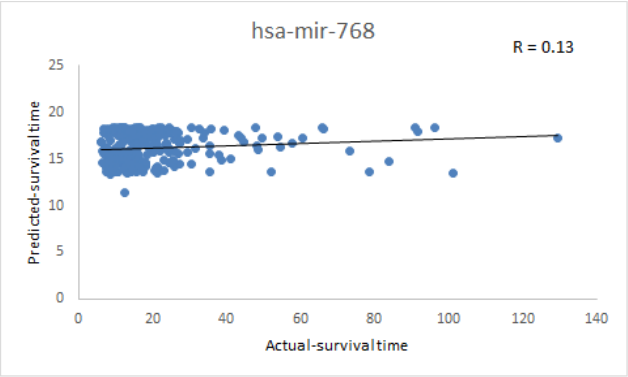

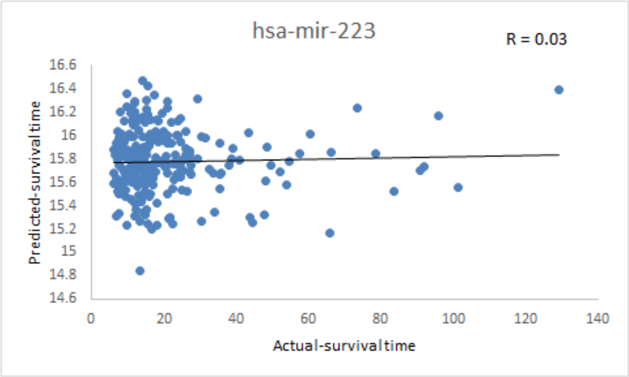

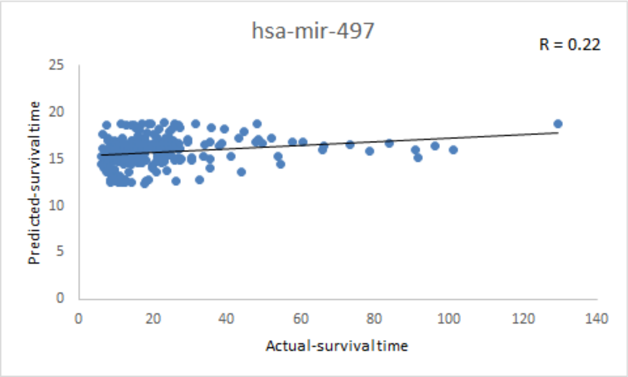

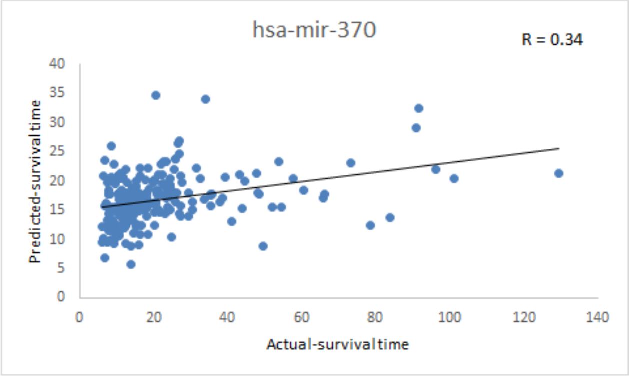

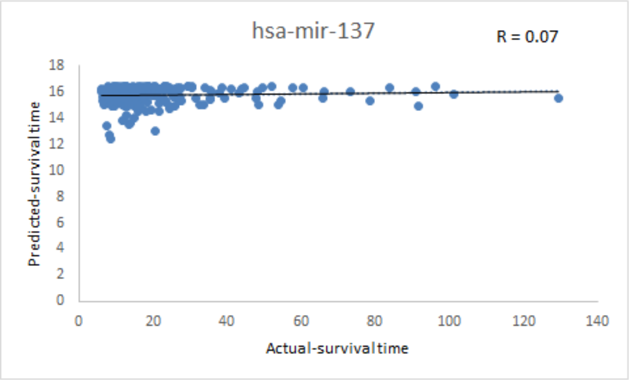

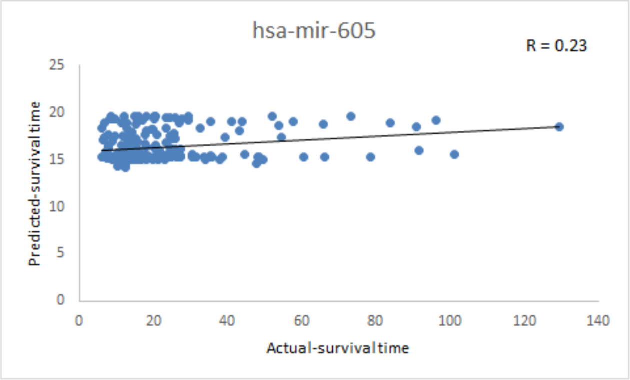

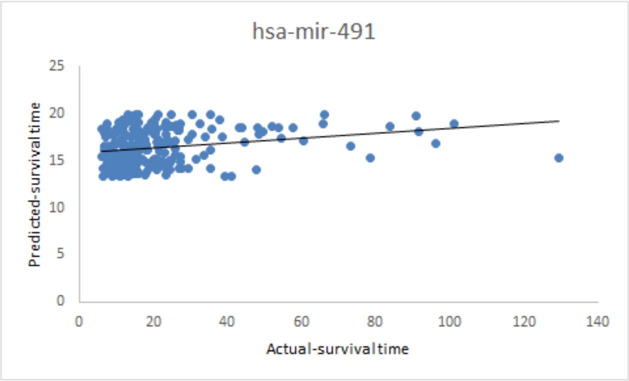

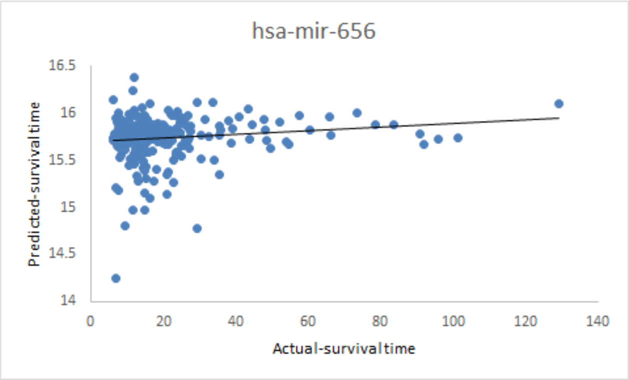

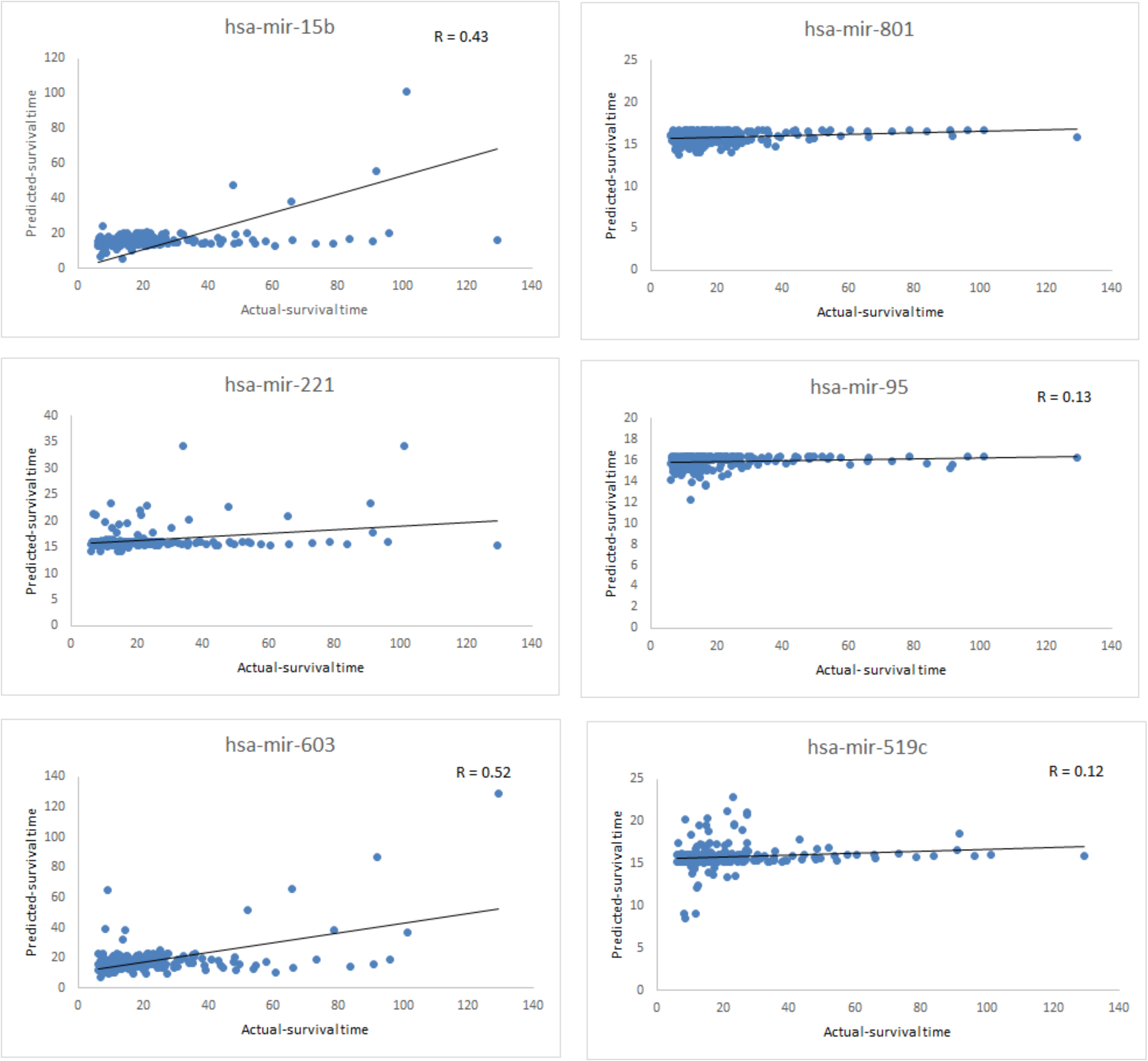

Figure S1. Individual effect of miRNA on survival time estimation. Correlation plots for 14 miRNAs**.**

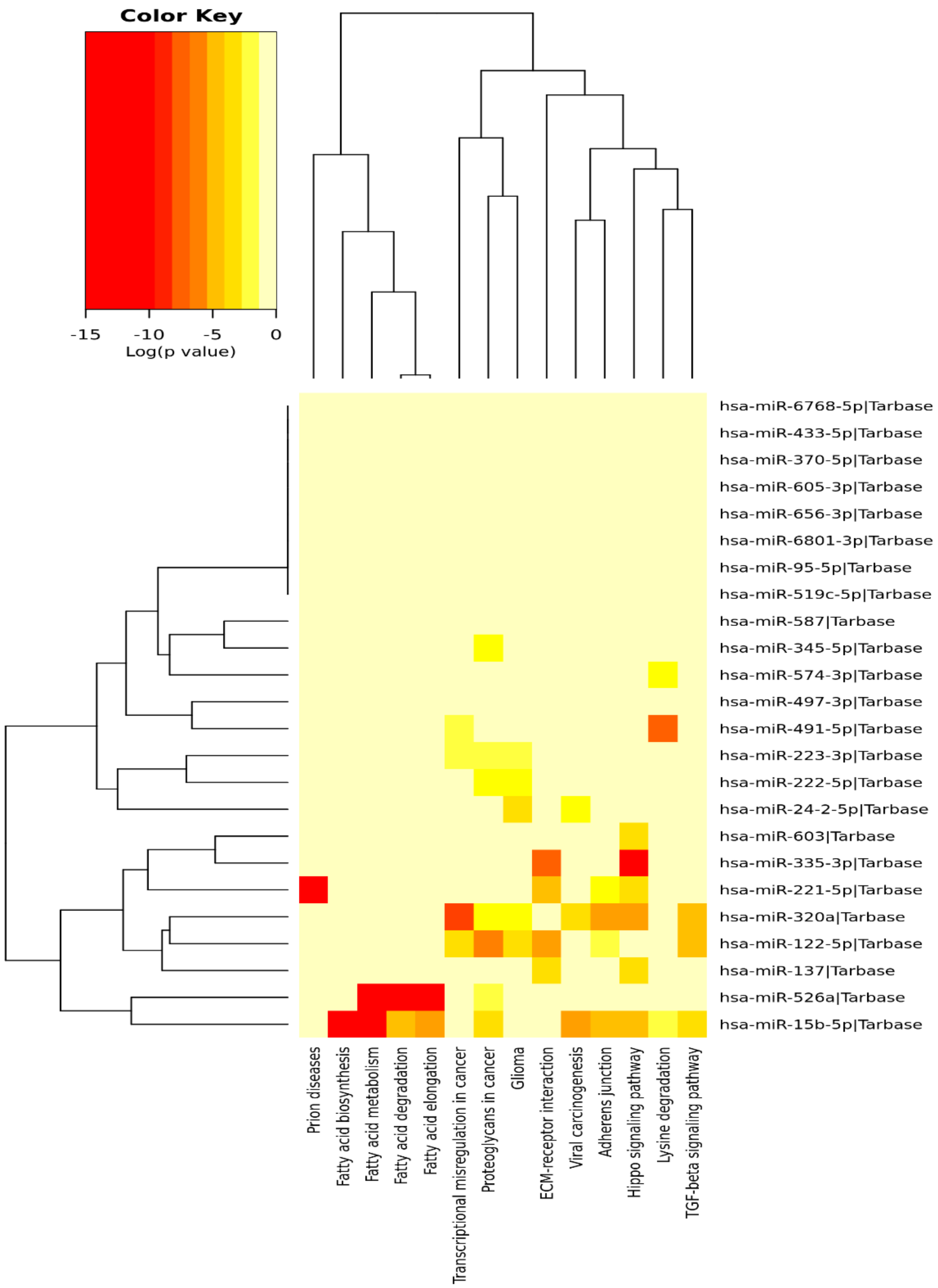

Figure S2. Heat map of the KEGG pathway. Identified 24miRNA signatures involved in different cancer pathway and signaling pathways.

Table S1. 24 miRNAs and their gene enrichment in the KEGG pathway

KEGG pathway	genes	miRNAs	p-value
Hippo signaling pathway	60	6	2.09E-13
Fatty acid metabolism	14	2	3.21E-10
Prion diseases	5	1	3.41E-10
Proteoglycans in cancer	90	7	5.81E-08
Fatty acid elongation	6	2	3.57E-06
ECM-receptor interaction	25	4	8.21E-06
Transcriptional misregulation in cancer	64	4	0.0001976
Adherens junction	42	4	0.0018324
Viral carcinogenesis	79	3	0.0030221
Fatty acid degradation	9	2	0.0030493
Lysine degradation	15	3	0.0039540
Glioma	26	5	0.0063682
TGF-beta signaling pathway	40	3	0.0216126
Fatty acid biosynthesis	4	1	0.0263490
Chronic myeloid leukemia	34	3	0.0627168
Pathways in cancer	126	3	0.0813562
Steroid biosynthesis	3	3	0.1139926
Pancreatic cancer	29	3	0.1949532
Metabolism of xenobiotics by cytochrome P450	3	2	0.2086712
Prostate cancer	43	4	0.269166
Hepatitis B	42	3	0.3719035
Signaling pathways regulating pluripotency of stem cells	43	2	0.3852171
Endocytosis	30	2	0.4068553
Protein processing in endoplasmic reticulum	59	3	0.5508437
Thyroid hormone signaling pathway	54	3	0.6208823
Colorectal cancer	23	2	0.6357839
Non-small cell lung cancer	18	2	0.6575763
Other types of O-glycan biosynthesis	11	3	0.6954478
Central carbon metabolism in cancer	24	2	0.7003775
Small cell lung cancer	23	1	0.782133
Shigellosis	14	2	0.799527
Melanoma	33	3	0.8393691
Regulation of actin cytoskeleton	23	1	0.8436391
Cell cycle	46	2	0.8831507
Oocyte meiosis	30	1	0.9047095
RNA transport	33	2	0.9106848
Bacterial invasion of epithelial cells	10	1	0.9193271
p53 signaling pathway	24	2	0.9354844
Spliceosome	24	2	0.9402877

Epstein-Barr virus infection	50	1	0.941338
FoxO signaling pathway	31	2	0.9447037
Estrogen signaling pathway	23	2	0.9634282
Bladder cancer	4	1	0.9884321
Long-term depression	10	1	0.9913877
mRNA surveillance pathway	7	1	0.9943654
Insulin signaling pathway	31	1	0.994509
AMPK signaling pathway	25	2	0.997179
Thyroid cancer	7	1	0.998155
Fc gamma R-mediated phagocytosis	15	1	0.999519
Vasopressin-regulated water reabsorption	4	1	0.999537
HIF-1 signaling pathway	7	1	0.9995399
Antigen processing and presentation	5	1	0.9997231
Biosynthesis of unsaturated fatty acids	2	2	0.9997589
RNA degradation	8	1	0.9999831
mTOR signaling pathway	4	1	0.9999834
Glycosaminoglycan biosynthesis - keratan sulfate	1	1	0.9999858
Sulfur relay system	2	2	0.9999893
Adipocytokine signaling pathway	13	1	0.9999949
Glycosaminoglycan biosynthesis - chondroitin sulfate / dermatan sulfate	5	1	0.9999955
Salmonella infection	14	1	0.9999959
Ubiquitin mediated proteolysis	31	1	0.999997
Huntington's disease	2	1	0.9999984
Cocaine addiction	8	1	0.9999999
Allograft rejection	3	1	0.9999999
Valine, leucine and isoleucine degradation	1	1	1
Valine, leucine and isoleucine biosynthesis	1	1	1
Sulfur metabolism	1	1	1
RNA polymerase	12	1	1
Renin-angiotensin system	1	1	1
Intestinal immune network for IgA production	2	1	1
Inflammatory bowel disease (IBD)	3	1	1
